# P-623. Development and clinical evaluation of a rapid diagnostic tool for point-of-need detection of Nipah virus

**DOI:** 10.1093/ofid/ofaf695.836

**Published:** 2026-01-11

**Authors:** Mohammad Enayet Hossain, Akash Saha, Prakash Ghosh, Arianna Ceruti, Jenifar Quaiyum Ami, Subyeta Binte Sarwar, Wasik Rahman Aquib, Tahmina Shirin, Christina Spiropoulou, Joel M Montgomery, Syed Moinuddin Satter, Ahmed Abd El Wahed, Mohammed Ziaur Rahman

**Affiliations:** icddr,b (International Centre for Diarrhoeal Disease Research, Bangladesh), Dhaka, Dhaka, Bangladesh; icddrb, Dhaka, Dhaka, Bangladesh; Leipzig University, Dhaka, Dhaka, Bangladesh; Leipzig University, Dhaka, Dhaka, Bangladesh; icddr,b, Dhaka, Dhaka, Bangladesh; icddrb, Dhaka, Dhaka, Bangladesh; icddr,b (International Centre for Diarrhoeal Disease Research, Bangladesh), Dhaka, Dhaka, Bangladesh; Institute of Epidemiology, Disease Control and Research (IEDCR), Dhaka, Dhaka, Bangladesh; US CDC, Atlanta, Georgia; Centers for Disease Control and Prevention (CDC), Atlanta, Georgia; icddr,b (International Centre for Diarrhoeal Disease Research, Bangladesh), Dhaka, Dhaka, Bangladesh; University of Leipzig, Leipzig, Berlin, Germany; icddr,b (International Centre for Diarrhoeal Disease Research, Bangladesh), Dhaka, Dhaka, Bangladesh

## Abstract

**Background:**

Nipah virus (NiV) is a bat-borne, highly pathogenic, stage III emerging zoonotic virus, with more than 70% case fatality rate in Bangladesh. As no treatment or vaccine is available, the rapid diagnosis of suspected cases and specific control measures are imperative for the fast containment of the disease. Current diagnostic methods for NiV, including RT-qPCR and ELISA, are time-consuming and often inaccessible in resource-limited settings. Therefore, development of a fast, sensitive and field-deployable diagnostic tool has been a pressing demand.

Diagnostic performance of RT-RAA assay

**Figure:**
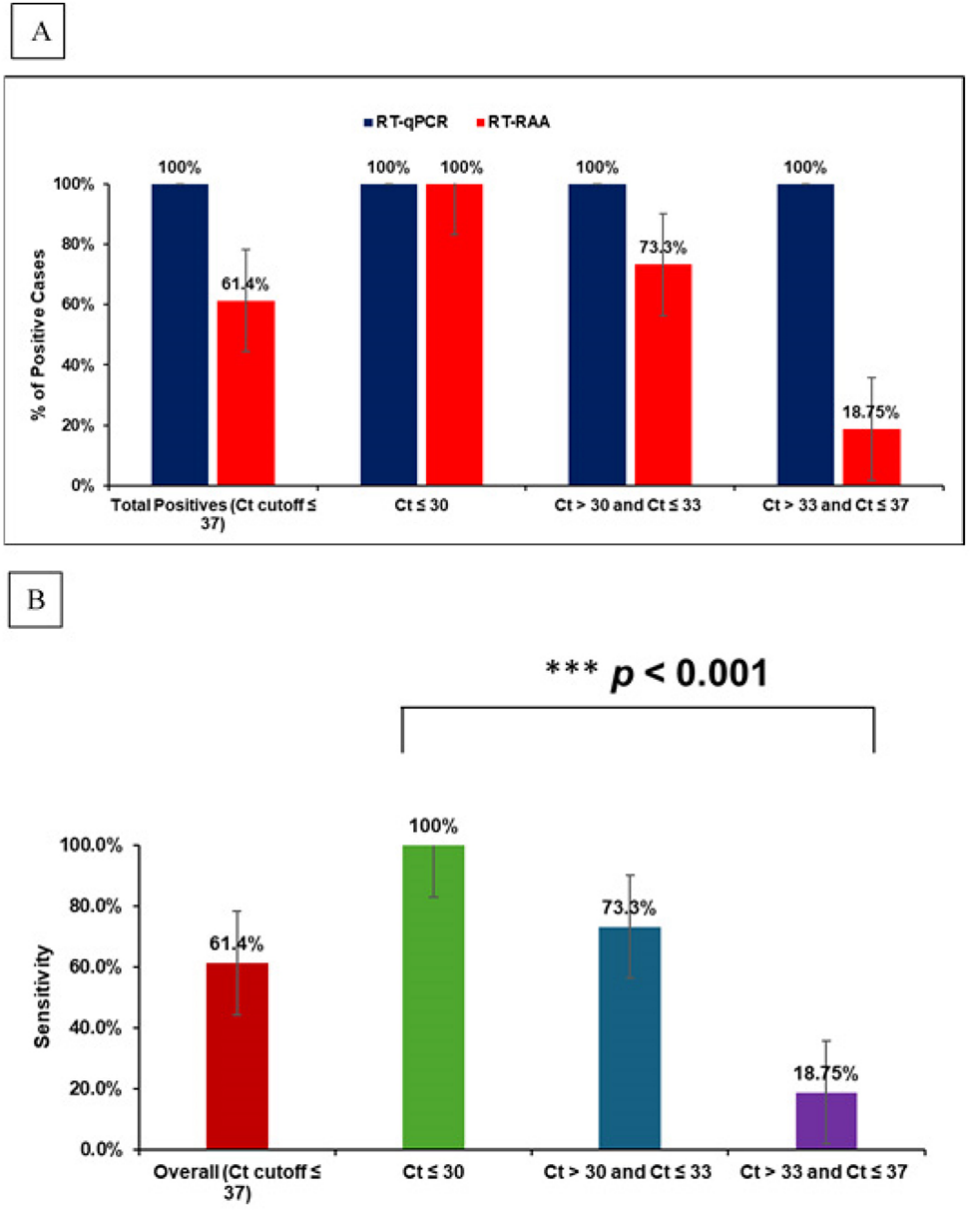

Performance of RT-RAA assay compared to RT-qPCR in detecting NiV in clinical samples stratified by Ct value ranges. (A) Overall positivity rate of Nipah virus in 90 clinical samples as detected by RT-RAA and RT-qPCR assays. (B) Sensitivity of NiV RT-RAA assay in different cases. The chi-square test of independence revealed a highly significant association between Ct value and RT-RAA assay sensitivity (p-value < 0.001).

**Methods:**

We developed and validated an isothermal, fluorescent-based, reverse-transcription recombinase-aided amplification (RT-RAA) assay for rapid and successful detection of NiV in clinical samples. Different primers and a probe were designed targeting the conserved region of the nucleocapsid (N) gene, and the optimal combination and reaction conditions were determined based on the RT-RAA efficiency. The performance of the assay was evaluated using 90 archived human clinical specimens collected during Nipah surveillance in Bangladesh. All samples were tested in parallel using both the developed RT-RAA assay and the gold-standard RT-qPCR.

**Results:**

The RT-RAA assay showed rapid detection of NiV at 42°C within 15 minutes, with a detection limit of 50 gene copies/reaction and no cross-reactivity with other pathogens. Of the 90 clinical specimens tested, 44 (48.9%) were confirmed NiV-positive by RT-qPCR. Stratifying cases by Ct thresholds revealed 29.5% as acute (Ct ≤ 30), 34.1% as subacute (30< Ct ≤ 33), and 36.4% as late-stage (33< Ct≤ 37) cases. The RT-RAA assay yielded 61.4% sensitivity, 100% specificity, and 81.1% diagnostic accuracy (Cohen’s Kappa = 0.62), with the highest sensitivity in acute cases (100%), moderate in subacute (73.3%), and lowest in late-stage cases (18.8%).

**Conclusion:**

The developed RT-RAA assay offers a rapid, accurate, and field-deployable diagnostic alternative for early-stage detection of NiV infection. Its excellent performance in detection of acute NiV infection can be exploited towards facilitating the rapid outbreak response, transmission breakdown, and improved clinical management in resource-limited settings.

**Disclosures:**

All Authors: No reported disclosures

